# Machine Learning Identifies Sexual Behavior Subgroups Among Men Who Have Sex with Men in Switzerland

**DOI:** 10.1007/s10508-025-03187-2

**Published:** 2025-07-07

**Authors:** Luisa Salazar-Vizcaya, Dunja Nicca, Vanessa Christinet, Roger D. Kouyos, Florian Vock, Sara Andresen, Andreas Lehner, David Haerry, Huldrych F. Günthard, Axel J. Schmidt, Andri Rauch

**Affiliations:** 1 Department of Infectious Diseases, Inselspital Bern University Hospital, University of Bern, Anna-Seiler-Haus, Geschoss J, 3010 Bern, Switzerland; 2 Epidemiology, Biostatistics and Prevention Institute, University of Zurich, Zürich, Switzerland; 3 Checkpoint-VD (PROFA Foundation), Lausanne, Switzerland; 4 Department of Infectious Diseases and Hospital Epidemiology, University Hospital Zurich, University of Zurich, Zurich, Switzerland; 5 Institute of Medical Virology, University of Zurich, Zurich, Switzerland; 6 Swiss AIDS Federation, Zurich, Switzerland; 7 Positive Council, Zurich, Switzerland; 8 Communicable Diseases Division, Swiss Federal Office of Public Health FOPH, Bern, Switzerland; 9 Department of Public Health, Environment and Society, London School of Hygiene and Tropical Medicine, London, UK

**Keywords:** Sexual behavior, Machine learning, Men who have sex with men, Group sex, Sexual orientation, HIV

## Abstract

Sexual behavior is heterogeneous and dynamic. Characterization of such complexity constitutes evidence for public health authorities and caregivers concerned with the framing of sexual health messages aimed at specific subgroups. We developed a machine-learning-based methodology for inference and characterization of such subgroups from longitudinal data on men who have sex with men (MSM) attending individual sexual health counseling sessions. Because longitudinal data take time to record, we assessed the ability of first visit data to predict subgroups’ membership. Our methodology comprised two main steps: (1) Hierarchical clustering to group 2349 HIV-negative MSM based on their self-reported longitudinal sexual behavior during visits to Swiss sexual health counseling centers between November 2016 and April 2019; and (2) Random forest-based classification to predict subgroup membership from first visit data. We found six subgroups with significant differences in behavioral trends, most of which sharply deviated from the overall trends. Two subgroups, which contained 37% of the study population, accounted for over 70% of the overall increases in condomless anal intercourse with non-steady partners, group sex, and having more than five anal intercourse partners. Subgroup-specific trends in online-dating and group sex were heterogeneous with opposing trends across subgroups. Data from first visits predicted trends of sexual behavior with accuracy ranging from 64 to 86%. This study evidenced specific sexual behavioral subgroups that might benefit from customized sexual health messages, demonstrated that first visit registries could predict subgroups, and contributes an algorithmic alternative for establishing subgroups relevant to inform customized sexual health messages that capture sexual behavioral diversity.

**Supplementary Information:**

The online version contains supplementary material available at 10.1007/s10508-025-03187-2.

## Introduction

Sexual behavior vary across individuals and may change over time. Incomplete understanding of heterogeneity in sexual behavior may expose sexual health care, public policy and research to the influence of potentially oversimplified interpretations of summary data and of potentially misleading stereotypical representations of people, especially sexual minorities. Conversely, algorithmic and comprehensive characterization of such heterogeneity, allowing for the identification of subgroups with similar behavioral patterns might constitute key knowledge to deliver individual sexual health messages likely to resonate with people’s interest and needs. Such knowledge could also contribute context to health-care-provided personalized counseling on sexual health. For instance, messaging tailored to well-defined behavioral groups could significantly enhance the impact of interventions aimed at reducing STI transmission by promoting, where relevant, reduced exposure, regular testing and treatment, partner notification, or access to peer group support.

Detailed characterization of behavioral heterogeneity across time and within individuals requires also detailed information on individuals’ characteristics, behaviors and changes in such behaviors. Voluntary counseling and testing (VCT) centers across Switzerland offer personalized counseling on sexual health and are also enabled to collect data on sexual behavior prior to the counseling session. VCT counseling takes place in personal sessions with highly trained health-care workers, and focuses on prevention of sexually transmitted infections (STIs), as well as on STI-testing(*Checkpoint*). VCT centers were at the core of a recent national cohort set to study common STIs among multi-partner men (Schmidt et al., 2020) and women (Vernazza et al., 2020). Since 2008, an increasing number of these VCT centers have adopted an online tool developed and provided by the Swiss Federal Office of Public Health. The tools’ main objective is to support individual counseling sessions by gathering information on sociodemographic characteristics, previous vaccinations, diagnosed STIs, sexual behavior, and drug use. This type of information is often easier to assess with this anonymous online tool than in face-to-face interviews. During VCT counseling sessions, the tool also provides up-to-date information on alternatives for medical support. The VCT centers have the option of providing their clients with anonymous but person-specific codes, which enable longitudinal follow-up (Schmidt et al., 2020). However, a minority of centers have used this alternative consistently. We studied the data from men who have sex with men (MSM) with repeated visits to VCT centers. In contrast, most previous studies assessing sexual behavior among MSM are either cross-sectional surveys (Marcus et al., 2012) or restricted to specific MSM subgroups (such as MSM taking PrEP or living with HIV) (Hovaguimian et al., 2022) (Scherrer et al., 2022). Additionally, information on longitudinal changes in sexual behavior among MSM in nationwide representative studies is very scarce.

In order to characterize heterogeneity in sexual behavior among MSM attending the aforementioned individual counseling sessions, we developed an intuitive machine-learning-based methodology for automated (1) inference of groups with similar time updated sexual behavior trajectories; (2) among those, identification of subgroups with behavioral trends that might warrant attention; and (3) early identification of members of those subgroups.

## Method

### Participants

VCT centers use the online tool since 2008, among them all five Swiss *Checkpoints* (sexual health centers with a focus on, yet not limited to gay, bi and queer men) that were active at the time of data retrieval, two university hospitals and three large cantonal hospitals. For this study, we extracted data from MSM from the 12 centers that had provided some or all of their clients with person-specific codes. We included only MSM who presented their anonymized, yet person-specific code for at least two visits between 2010 and 2019, including MSM participants of the Swiss STAR trial 2016–17 (Schmidt et al., 2020; Vernazza et al., 2020). Of note, the dataset used for analyses did not contain the original person-specific codes, instead a randomly assigned code.

### Measures

Recorded variables included sociodemographic characteristics, health data and data on sexual behavior. Further information on variables stored by the tool has been published elsewhere (Schmidt et al., 2020). For the present study, we considered the following variables: date of visit, demographic information including age, gender identity, sex at birth, nationality, time living in Switzerland, canton of residence, partnership status; health information such as sexual happiness, HIV status, history of diagnosed sexually transmitted infections, current symptoms of STIs; social behaviors such as online *vs*. offline acquisition of sex partners; sexual behaviors such as number of partners, anal intercourse and condomless anal intercourse partners in the previous 12 months (steady and non-steady), group sex, sexualized drug use, sexualized alcohol use, and “Chemsex” (defined here as using any of crystal methamphetamine, ketamine, GHB/GBL, or mephedrone; often or always when having sex (Giraudon et al., 2018); and “negotiated safety” (using condoms consistently with non-steady partners but not with steady partners).

### Procedure and Statistical Analysis

**Fig. 1:**
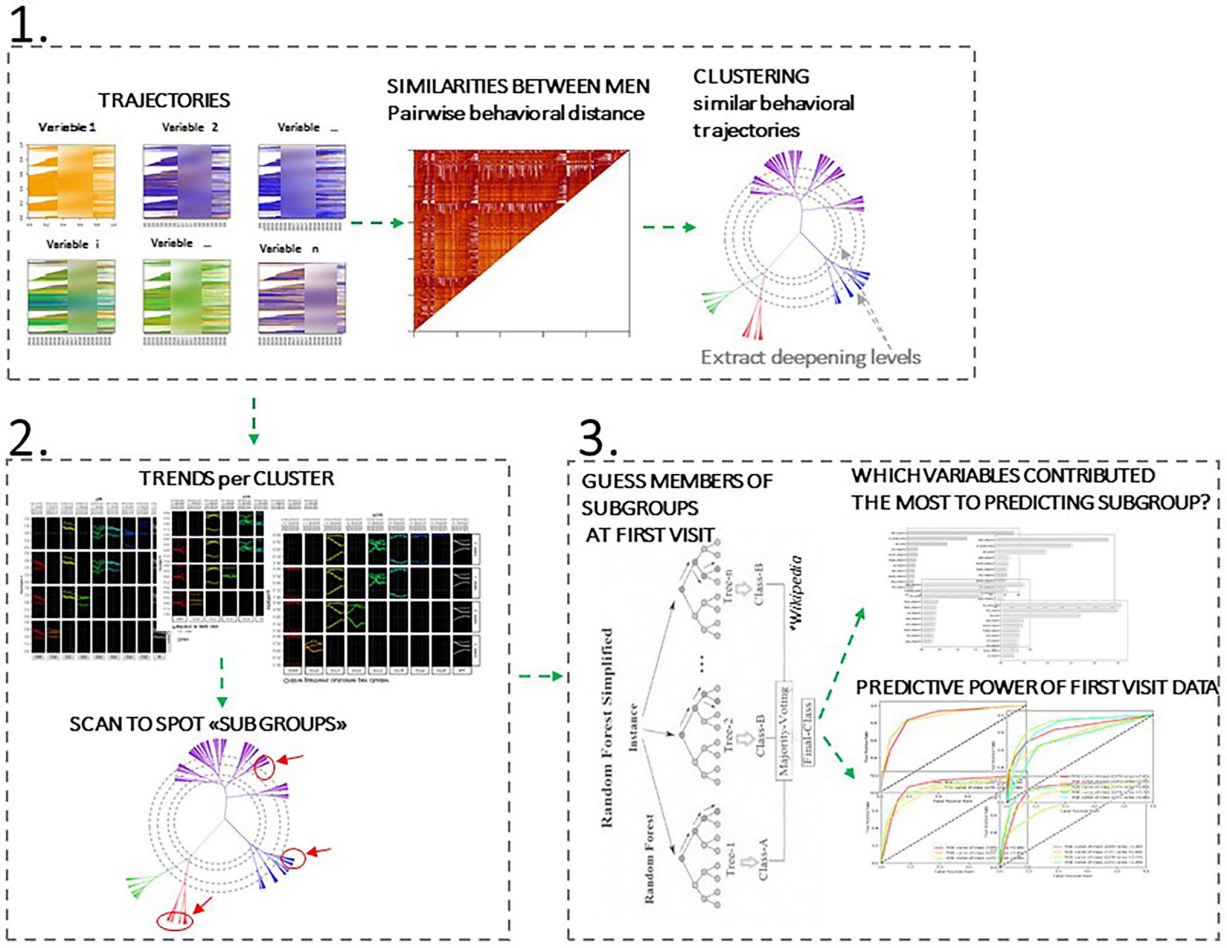
Schematic representation of the analytical pipeline to identify and predict subgroups. The pictures are intended to graphically illustrate the process and do not correspond to actual results

This study consists of three parts as depicted in Fig. 1: (1) recognition of patterns of change in sexual behavior over time, by clustering MSM according to their individual trajectories of sexual behavior; (2) identification of clusters whose changes and trends in sexual behavior might warrant attention (these clusters are termed subgroups from now on); and (3) identification of potential members of subgroups from first visits data only. In accordance, we defined two overlapping data groups: longitudinal behavioral data for parts (1) and (2), and first visit demographic and behavioral data for part (3). Supplementary Table S1 depicts the data available for this study.

#### Inclusion Criteria

*Longitudinal behavioral data for clustering:* Among candidates for clustering analyses, only records previous to any registered HIV diagnosis, in variables directly related with sexual behavior, recorded for more than 3 years, with frequency ever exceeding 20% in the overall population and with the number of valid records exceeding 10,000, were considered for clustering (“longitudinal variables,” Supplementary Table S1). To enhance robust clustering, we established a data density criteria.

*Data density criteria:* In order to enhance robust clustering of changes in sexual behavior, we established a clustering period based on availability of overlapping data registries between men, and selected men whose longitudinal data coverage exceeded the median coverage along that period. This process consisted of two steps: (1) definition of clustering “dense period,” by splitting the full-records time span in steps of one month and estimating for each of these time steps the number of men with at least one valid record for any of the longitudinal variables. The clustering period corresponded to the largest period along which this number exceeded the median over all time steps; (2) exclusion of men with insufficient data, by estimating the portion of the clustering period for which each man had at least one valid record for any of the longitudinal variables, and excluding those whose coverage was below the overall median.

*First visit data for early identification of clusters’ members:* These analyses included all available variables with at least 2000 valid first visit records, and complete cases among men whose longitudinal data were included for clustering.

### Identifying Patterns of Change in Sexual Behaviors

We defined behavioral trajectories as the succession over time of behavioral statuses in analogy with the behavioral matrices we previously described (Salazar-Vizcaya et al., 2020), with monthly update. The “behavioral status” of a man at a given point in time reflected his latest self-reported records concerning the longitudinal variables. For example, a man’s status could be as follows: engaging in condomless anal intercourse with non-steady male partners, having less than 5 anal intercourse partners, not engaging in Chemsex, engaging in group sex, not meeting partners online, not having a female sex partner.

To quantify dissimilarities between the behavioral trajectories of different men in our study population, we defined a metric. According to this metric, the behavioral distance between the pair of men labeled *j* and *k* is given by $\sum_{\upnu }{D}_{v}^{j,k}$, where ${D}_{v}^{j,k}=d{(M}_{v, \tau }^{j},{M}_{v, \tau }^{k})= {\omega }_{v}^{j,k}(1-\text{jaccard}{(M}_{v,\tau }^{j},{M}_{v,\tau }^{k}))$. Where “*jaccard*” refers to the similarity index of that name. The tensor-like ${M}_{v, \tau }^{i}$ expression represents the entry for behavioral variable *v* of the men indexed *j* at time $\tau ,$ e.g.,, it would equal 1 in behavioral variable “group sex” ($v$) for “John” ($i$) in January 2018 ($\tau$) if he reported having engaged in group sex at any point in January 2018. The weight ${\omega }_{v}^{j,k}$ accounts for the contribution of behavioral variable *v* to the distance by reflecting comparability between men with respect to *v*. This comparability measure grounds on the notion that the more overlapping valid records the more accurate a comparison is. The weight is therefore defined as:

$$ \omega_{v}^{j,k} : = \frac{{{\text{number}}\;{\text{of}}\;{\text{valid}}\;{\text{records}} \;{\text{for}}\;v\;{\text{ in}}\;{\text{ both,}}\;j\;{\text{and}}\;k\;\left( { = :\;{\text{overlaping}}} \right)}}{{{\text{max}}_{v} \;\left( {{\text{number}}\; {\text{of}}\; {\text{valid}}\;{\text{ records}}\;{\text{ in}}\; {\text{both}},\; j\;{\text{ and}}\; k\;{\text{ across}}\; {\text{all}}\; {\text{behavioral}} \;{\text{variables}}} \right)}} $$

### Clustering and Identification of Subgroups with Behavioral Trends in Sexual Behavior that Warrant Attention

We applied an unsupervised machine-learning method (agglomerative hierarchical clustering) to the matrix resulting from the pairwise comparison described above. Because agglomerative hierarchical clustering results in several, self-contained classifications of the population, each of which is determined by the level of the hierarchy one decides to consider, and we aimed at identifying clusters (subgroups) that warrant attention regardless of the choice of such level, we used an identification algorithm that iteratively screened the classifications that corresponded to the top 6 hierarchy levels. We therefore defined subgroups as those clusters in the hierarchy whose behavioral trends in any of the behavioral variables fulfilled the following set of heuristic criteria: (1) The frequency of the behavior must have reached or surpassed 25% within the cluster; (2) The frequency surpassed 75% over at least 95% of the study period (reflecting that the behavior in question is hegemonic) within the cluster, or the frequency changed (toward increased exposure to sexually transmitted infections) by at least 75% within the cluster. When clusters descending from larger (parent) clusters identified as subgroup also fulfilled the respective criteria, the decedents remained subgroups only if the second criterion differed in any way between parent and descendant. Descendant clusters were discarded otherwise (e.g., a descendant cluster was attained when a given behavior was hegemonic but not significantly increased in the parent cluster, while it was significantly increased in the descendant cluster).

To avoid jumps resulting from small denominators, in this step we discarded all segments of the trajectories for which the number of valid registries were below the first quartile along the full trajectory.

All algorithms were implemented in R 3.62 using *ape* (Paradis & Schliep, 2019).

*Early identification of subgroup members:* Behavioral clusters were inferred using longitudinal data exclusively. Because longitudinal data for classification among subgroups is not available in many instances, and because collection of longitudinal data as part of routine-counseling and –testing is costly and time-consuming, we sought to anticipate upcoming changes in sexual behavior by identifying likely members of subgroups without requiring longitudinal data. To do this, we trained a supervised machine-learning algorithm to allocate men across clusters (random forest classifier) using men’s first VCT visit data only (i.e., sociodemographic and unrelated/related with sex), as detailed in Text S1. These analyses were performed in Python version 3.7.6 (Van Rossum & Drake, 2009), Tensor flow (Abadi et al., 2016), Pandas (McKinney, 2011) and included two types of non-longitudinal variables: non-explicitly related to sex (e.g., demographics) and first visit records for sexual behavior (i.e., online dating, group sex and Chemsex). Only variables with valid records for at least 2000 men were included in this part of the analyses.

## Results

Before applying the inclusion criteria, the data available for this study comprised information collected during 17,680 counseling sessions with 4712 men who had at least two recorded visits between June 2010 and December 2019. These sessions took place in 12 different VCT across Switzerland.

### Overall Trends in Sexual Behavior

Variables suitable for longitudinal analyses (according to the density criteria) were on online dating, number of anal intercourse partners, condomless anal intercourse with non-steady partners (nsCAI), group sex and partnership status (i.e., single or in a steady relationship). After computing the behavioral trajectories for all available patients, 11,269 counseling sessions remained in the analyses. These sessions spanned between January 2017 and May 2019 and included 2349 MSM counseled in 12 VCT centers across three main geographical regions.

**Fig. 2:**
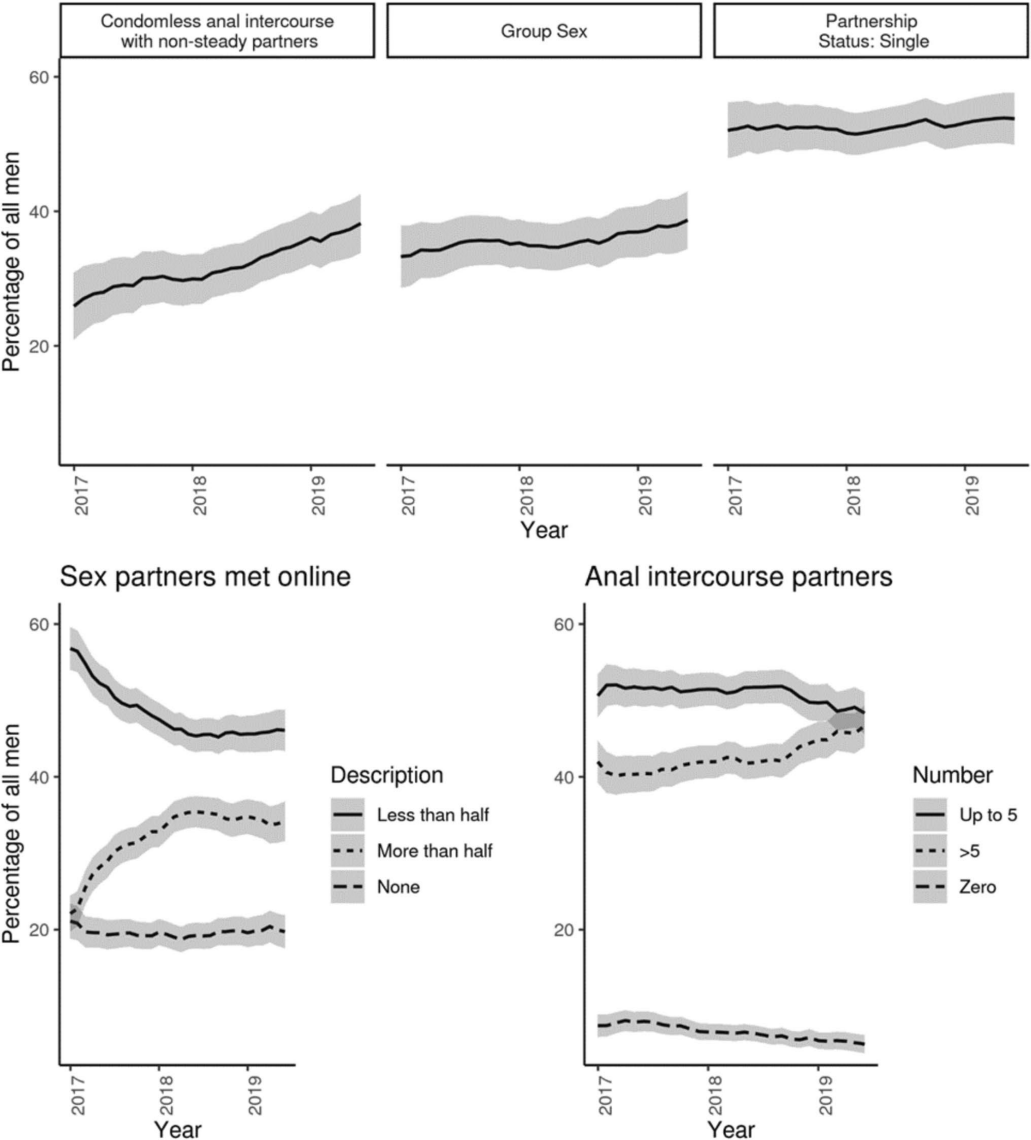
Overall trends In sexual behavior. Percentage of the study population reporting the sexual behavior. Sex partners met online were summarized as None, Up to half vs More than half (see also Supplementary Table S1 for a detailed description of the variables)

The age of men included in the analyses ranged from 16 to 84 years in the first visit. Table 1 shows the baseline characteristics of men included in the analyses. Overall nsCAI increased over the study period from 25.9% (95%-ci:20.9–30.9) to 38.2% (33.8–42.7; Fig. 2). The frequency of group sex also increased although at a lower speed (33.3% (28.7–37.9) to 38.7% (34.4–43.0)) (Fig. 2). Partnership status was the most stable variable we observed in this study with half of MSM reporting to be single (between 52.0% (47.9–56.2) and 53.8% (49.9–57.6)).

**Table 1:** Characteristics of the men who have sex with men included in the analyses

Number of men	2349
Number of visits	11,269
Age at first visit (years)	36 (IQR: 28–44)
Years covered (range; IQR)	2015–19; (2017–18)
*Country of origin*	
Switzerland	68.4%
Neighboring countries^a^	13.8%
other	17.8%
*Region in Switzerland*^*b*^	
Zurich region	17.6%
Lake Geneva region	55.3%
Other Swiss regions	27.1%
Sexual behavior (ever reported during follow-up)	
*Number of male anal intercourse partners*^*c*^	
> 5	76.5%
Up to 5	53.6%
*Partners met online*	
None	26.7%
About half, Less than half, or Yes	57.7%
More than half or Nearly all of them	41.8%
Group sex^c^	48.0%
Condomless anal intercourse with non-steady partners (nsCAI)^d^	58.8%
Partnership status single	64.2%

See Supplementary Table S1 for a detailed description of the variables

^a^Neighboring countries: Austria, Germany, France and Italy; ^b^ *Zurich region* accounts for Canton Zurich (1,564,662 inhabitants by the end of 2021; Lake Geneva region includes Cantons Geneva, Vaud and Valais (1,685,625) and the complementary regions for the remaining 5,488,504) (Swiss Federal Statistics Office. STAT-TAB - interactive tables, 2021); ^c^in the previous 12 months; ^d^in the previous 3 months

Online dating increased over the study period: MSM were increasingly more likely to move from reporting that they met less than half or about half of their sex partners online to reporting that they met either more than half or nearly all their sex partners online (Fig. 2).

MSM became more likely to report having had more than 5 anal intercourse partners in the previous 12 months over the study period (final record: 46.6% (43.9–49.4). By the end of the study period reporting more than 5 anal intercourse partners was nearly as common as reporting up to 5 anal intercourse partners (48.3% (45.6–51.1%). Supplementary Table S1 shows the numbers of records available for analyses as well as the coding for variables outcome grouping.

### Automated Identification of Subgroups

**Fig. 3:**
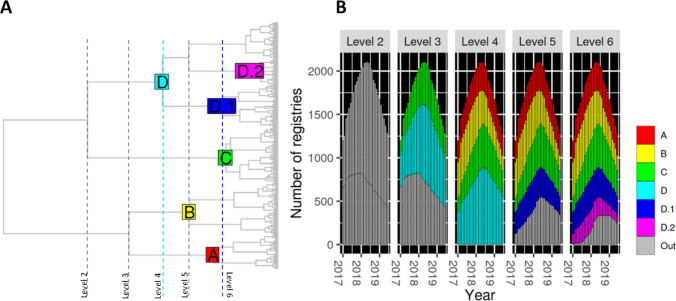
Clustering patterns and identified subgroups. **A)** Clustering pattern, hierarchy levels scanned and subgroups identified (labeled, colored nodes). **B)** Size of subgroups. “Out” accounts for clusters that did not meet the subgroups criteria

**Fig. 4:**
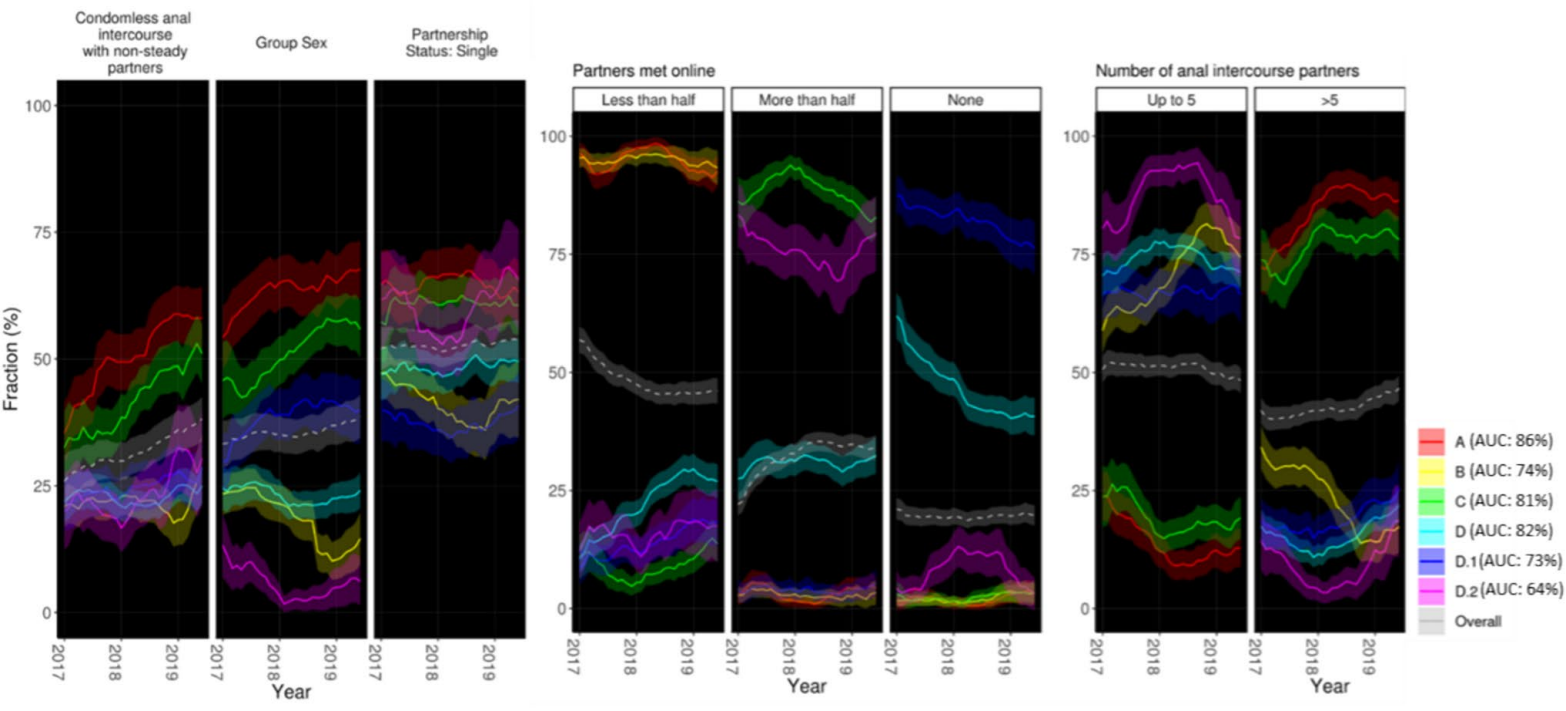
Trends in sexual behavior across subgroups. The gray dashed lines show the overall trends

The top 6 levels of the agglomerative hierarchical clustering pattern (Fig. 3) defined 10 clusters of which 6 fulfilled the subgroup’s criteria. These included two subclusters of the largest subgroup (D, 42% of the study population). Figure 3A locates the six subgroups in the hierarchy (A–D and D1, D2). The relative sizes of all clusters remained stable over the study period (Fig. 3B), and all members of the study population were in at least one subgroup. Table 2 shows the baseline characteristics of MSM across subgroups, and Fig. 4 displays their trends in sexual behaviors over time.

**Table 2:** Characteristics of the men who have sex with men per subgroups

	A	B	C	D	D.1	D.2
*N* = *2349*						
Number of men (n, %)	330 (14%)	494 (21%)	529 (23%)	996 (42%)	591 (25%)	244 (10%)
Median age at first visit (years, IQR)	36 (29–44)	34 (27–42)	34 (28–43)	35 (28–46)	33 (27–42)	33 (28–41)
Years covered (range; IQR)	2012–19 (2017–18)	2017–19 (2016–18)	2012–19 (2017–19)	2012–19 (2017–19)	2012–19 (2016–18)	2012–19 (2017–18)
*Country of origin*						
Switzerland	67.3%	70.6%	65.8%	69.0%	73.1%	63.5%
Neighboring countries^a^	13.3%	11.9%	15.1%	14.2%	15.6%	15.6%
*Region in Switzerland*						
Zurich region	9.1%	16.0%	23.4%	18.2%	11.6%	26.2%
Lake Geneva region	73.0%	65.2%	46.5%	49.1%	62.0%	43.0%
Other Swiss regions	17.9%	18.8%	30.0%	32.6%	26.4%	30.7%
*Sexual behavior (ever reported during follow-up)*						
*Number of male anal intercourse partners*^b^						
> 5	98.8%	75.1%	97.0%	58.9%	60.7%	61.5%
Up to 5	26.7%	69.0%	21.7%	71.8%	74.1%	75.8%
*Partners met online*						
None	11.8%	10.3%	6.2%	50.7%	88.6%	18.9%
Up to half	99.4%	99.4%	23.8%	41.2%	33.6%	35.7%
More than half	10.0%	8.3%	94.1%	41.2%	9.9%	86.1%
Group sex^b^	77.0%	39.3%	66.7%	32.7%	51.4%	15.2%
Condomless intercourse with non-steady male partners (nsCAI)^c^	81.5%	55.7%	72.6%	45.6%	47.9%	50.8%
Partnership status single	75.5%	60.3%	71.3%	58.7%	48.6%	67.2%

^a^Neighboring countries: Austria, Germany, France, and Italy

^b^in the previous 12 months; ^c^in the previous 3 month

Figure 4 displays the trends in sexual behavior across subgroups. In brief, Subgroup A had high frequencies of sexual behaviors associated with STI exposure (nsCAI, group sex and more than five anal intercourse partners in the previous 12 months) with most members reporting online-dating and being single. Subgroup B had high frequency of online-dating and showed a decline in the frequencies of group sex and number of intercourse partners over the study period. Subgroup C had high, yet decreasing rates of online-dating combined with also high and rising indicators of exposure to STI transmission. Subgroup D had low frequencies of sexual behaviors associated with STI exposure and sharply increasing frequencies of online-dating. Subgroup D1 had low, slowly increasing frequencies of online-dating and high frequencies of group sex. Subgroup D2 had low frequencies of group sex and of more than five anal intercourse partners in the previous 12 months, and met their sex partners predominantly online.

### Comparison Between Subgroups

Subgroup A (14% of the study population) was characterized by the highest frequencies of sexual behaviors associated with STI exposure across the hierarchy. The frequency of nsCAI, group sex and sexual contacts with more than five anal intercourse partners in the previous 12 months was highest and increased in this subgroup over time. Most members of this cluster consistently reported having met up to half of their partners online (range: 91%–98%). MSM in this subgroup were predominantly single.

Subgroup B (21%) shares the predominance and persistence of online-dating (range: 92%–96%) with subgroup A (both share parent cluster Fig. 3A). Subgroup B however had an evident decline in the frequencies of group sex and number of anal intercourse partners over the study period.

Subgroup C (23%) had the most active, yet decreasing, involvement in online-dating. The frequencies of “More than half of partners met online” were highest in this cluster, ranging from 82 to 94% over the study period. The frequencies of nsCAI and group sex were almost as high and increased almost as fast as those observed in Subgroup A. Such was also the case of more than five anal intercourse partners in the previous 12 months, which plateaued after the first year. MSM in this subgroup were predominantly single (Table 2).

Subgroup D (42%) comprises a segment of the study population with relative low frequencies of sexual behaviors associated with STI exposure, i.e., nsCAI and more than five anal intercourse partners, most of whom were increasingly active in online-dating. The frequency of reporting having met up to half of their partners online tripled over the study period, while the rates of nsCAI and group sex remained significantly lower than those in the general population. Similarly, the number of individuals reporting more than five anal intercourse partners in the previous 12 months, which began increasing in 2018, also remained below general population levels.

Subgroup D.1 (25%) was characterized by the lowest frequencies of online-dating in the whole population. It also had the highest frequencies of group sex among D subclusters. The vast majority of its members consistently reported not having met sex partners online over the study period (range: 76%–88%) and few ever reported having met more than half of their partners online (9%). However, in resemblance of its parent subgroup (subgroup D), a consistently increasing number of members of this group reported having met up to half of their sex partners online.

Subgroup D.2 (10%) was characterized by the lowest frequencies of group sex (it even declined over the study period) and of more than five anal intercourse partners in the previous 12 months. Members of this subgroup found most of their sex partners online with a frequency of “more than half of sex partners met online” that ranged between 69 and 83% over the study period. Despite below-average nsCAI levels, this subgroup displayed steeply increasing frequencies of this item since 2018.

Early identification of subgroup members Figs. 4 and Supplementary Figure S1 show the accuracy of the prediction, which ranged between 64% for subgroup D2 and 86% in subgroup A. The classifier was most sensitive to first visit records on online dating, age, and the number of anal intercourse partners in the previous 12 months (Supplementary Figure S2).

## Discussion

We combined pattern recognition techniques to study self-reported data on sexual behavior from HIV-negative MSM in Switzerland with repeated visits to sexual health counseling centers between January 2017 and May 2019, including 12 VCTs across the country. Our study evidenced heterogeneous configurations of sexual behavior that were masked by the overall trends. The differences between the six subgroups we identified were striking, and in evident discrepancy with overall trends in nsCAI, group sex, partnership status, online-dating, and the number of anal intercourse partners in the previous 12 months. Data from first visits predicted trends of sexual behavior over the study period with accuracy ranging from 64 to 86% across subgroups.

Behavioral trends “refracted” by the algorithms deployed in this study could indicate that trends in sexual behavior emerge from heterogeneous decision-making on sexuality taking place in the study population. The consequences of such hypothesized heterogeneity are however invisible to overall trends, while evident for the methodology we present in this manuscript. For instance, none of our subgroups resembled the overall trend in reports of more than 5 anal intercourse partners in the previous 12 months. Moreover, we found striking differences across subgroup-specific trends in this sexual behavior. Importantly, overall trends were insensitive to recent, fast behavioral changes in the smallest subgroups (e.g., reductions in number of anal intercourse partners in subgroup B or group sex on subgroup D.1). Online-dating also exemplifies large differences between overall trends and subgroup-specific trends as well as heterogeneity across subgroups: while the overall fraction of men who do not meet sex partners online remained stable at about 20% over the study period, the members of a subgroup containing a quarter of the population (subgroup D.1) who were initially unexperienced in online dating, became twice as likely to report having met up to half of their partners online toward the end of the study period.

Overall percentages of nsCAI, online dating, group sex, and the number of anal intercourse partners increased over the study period, which suggest increased STI exposure in the overall MSM population. These increases would also indicate the need for interventions that combine the dissemination of sexual health messages that highlight the subsequent risk of STIs, as well as options to mitigate such risk. Examples of available options to prevent STI include screening and partner notification, prescriptions of medicaments like post-exposure doxycycline for syphilis and chlamydia prevention (Luetkemeyer et al., 2023) and vaccination (e.g., against Hepatitis B and potentially against gonorrhea in the near future (Abara et al., 2022)). Our results indicate and illustrate why, if implementation is feasible, customized preventive measures (e.g., combining preventive measures with sexual health messages) that focus on segments of the population with similar behavioral patterns might be more efficient than those addressing the overall MSM population. For example, the machine-learning-based method we propose could help prioritize and customize interventions to focus on subgroups A and C and provide information on STI prevention and sexual health counseling in relation with nsCAI with multiple partners and group sex, for instance, in the context of sexual parties. These two subgroups account for only 37% of the population, but accounted for more than 70% of the increases in nsCAI, group sex, and having more than five anal intercourse partners.

Our study showed potential differences in the needs and interests of online-dating App users. For instance, the subgroup with rapidly increasing frequencies of online dating (subgroup D.1) could benefit from information on sexual health suited to persons who are new to this practice. However, this subgroup was also characterized by low frequencies of nsCAI and by low numbers of anal intercourse partners. This suggests that, irrespective of the means of communication, general STI prevention messages (e.g., condom use) might not be as relevant for members of subgroup D1 as they would for other dating app users in subgroups C and D2. Moreover, information for starting to use dating apps appears to be largely irrelevant for the rest of the population as they are experienced in the use of such tools. Furthermore, in defiance of possible preconceptions, our results suggest that online-dating is not necessarily an indicator of increased STI exposure. For instance, most members of the group with the least potential exposure to STIs in the whole study population (D.2.: below-average nsCAI, rarely reporting group sex and least likely to report that they had more than five anal intercourse partners in the previous 12 months) were also consistent in reporting that they found most or all of their sex partners online (range: 69%–83%). This is in line with a study on MSM in Amsterdam which did not find significant differences in condom use between offline- and online-dating sexual encounters with casual partners (Heijman et al., 2016).

The widespread use of social media and dating apps could be used to deliver sexual health messages tailored to well-defined behavioral groups (Kesten et al., 2019). In principle, social media feeds and personal messages in apps could serve as transmission channels. However, dating apps and social media bear concerns regarding private data sharing. Furthermore, overcoming barriers for the materialization of such cooperation between public health authorities and social media/dating apps might be challenging (Castellanos-Usigli, 2022). A pragmatic approach to overcome this barrier could be to present users who agree to receive sexual health messages with the possibility of choosing between alternative descriptions of sexual behavior designed to match subgroups. Sexual health messages tailored to the respective subgroup would follow the user’s choice. Our results point to two subgroups likely to be reached by means of dating apps (C and D.2). These two groups have seemingly opposite sexual behavior, yet are by far the most likely to meet most of their anal intercourse partners online.

In contradiction with potential preconceptions that automatically link group sex and engagement in other sexual behaviors known to be associated with STI exposures, our study suggested disconnections between group sex, nsCAI and the number of anal sex partners. For instance, we identified a subgroup with > 30% frequency of group sex (slightly above the overall average) and significantly below-average frequency of nsCAI and number of anal intercourse partners. Previous studies have also found diverse combinations of sexual behaviors deriving in differences in STI exposure among MSM engaging in group sex (Smith et al., 2019; van den Boom et al., 2016).

Our study contributes detailed insights on sexual behavioral heterogeneity among HIV-negative MSM, as well as to the understanding of the risk of oversimplification that derives from summarizing sexual behavioral data from large populations. We did this by using unsupervised and supervised machine-learning techniques on data that originated in VCT centers across Switzerland. The high level of detail achieved in a VCT counseling session, and the subsequent possibility to accurately address relevant topics (i.e., clients can communicate their concerns, interests and experiences directly to trained health-care workers) is however inheritably superior than that of to our algorithms, which could not outperform a counseling session. Our results could however help contextualize a client’s sexual behavior to the light of ongoing trends. Automated classification of a client among subgroups at first visit could be made available to VCT counselors in centers using a person-specific code. They could use it strategically to raise complementary, potentially relevant topics hinted by the predicted subgroup. Random forest classifiers naturally produce variables ranking according to their importance for the classification task. This feature allowed us to identify online-dating, age, and the number of anal intercourse partners in the previous 12 months as the top decisive variables for early identification of subgroups members.

Machine-learning has the potential to improve and complement existing public health policy by boosting the effect of prevention intervention strategies (Xiang et al., 2022). Our study enquired, by means of machine-learning methods, longitudinal data to then assess the potential of cross-sectional data to predict upcoming trends in sexual behavior. Most previous machine-learning approaches to address sexual behavior have mainly focused on cross-sectional data and transmission prediction. Supervised machine-learning approaches on large datasets are increasingly and often successfully explored as an alternative to increase the predictive power of models assessing the risk of HIV infections at the individual level (Bao et al., 2021; He et al., 2022). Latent class analysis (LCA) is an increasingly popular pattern recognition method in the study of HIV and STI prevention. A LCA on MSM attending centers for sexual health in the Netherlands identified classes with distinct combinations of sexual behaviors. In consistence with our results, their findings evidenced the need to address such groups separately (Slurink et al., 2020). A cross-sectional study in Amsterdam that used LCA on individual data on drug use during sex classified MSM in distinct classes. The classes with higher levels of drug use overlapped with also higher levels of exposure to and acquisition of STIs (Achterbergh et al., 2020). Another cross-sectional LCA from Hong Kong found increased risk of HIV transmission in medium and higher levels of drug use for sex (Wong et al., 2020). As opposed to LCA, hierarchical clustering does not require assumptions on the distribution of the data, a model fit or a predetermined number of clusters. Moreover, we implemented an algorithm able to explore deepening levels of the hierarchy in the search for subgroups with trends that might warrant attention. This approach ensured that the decision on the number of clusters was directly based on the research question.

### Limitations

The beginning of the algorithmically determined study period coincides with the release of guidelines by the Swiss Federal office of public health for the prescription of Pre-Exposure Prophylaxis (PrEP) as HIV-transmission prevention alternative (FCSTI, 2016). PrEP reduces the probability of HIV transmission during condomless anal intercourse, so this might have triggered an increase in nsCAI. Longitudinal data on this early stage of PrEP suitable for our study was however unavailable. Data on PrEP is collected in the frame of SwissPrEPared (Hovaguimian et al., 2022) since 2019 and in the updated VCT questionnaires since 2020. However, it could not be included in this study dataset as it closed in April 2019. Therefore, we could not assess the potential effect of PrEP in sexual behavior trends. Our study’s time period was constrained by limited availability of repeated measurements, which were only possible when centers applied person-specific identifiers. The initial data time span of 12 years was reduced to 30 months by applying the inclusion criteria for longitudinal behavioral data for clustering, thereby limiting the possibility to look at long-term trends in sexual behavior. Attainment of such identifier codes is not broadly practiced in all VCT centers, which could have led to unmeasured selection bias in sites that do not systematically use unique identifier codes. Increased use of this identifier in all sites would boost the precision of analyses of the kind presented in this manuscript and the prediction power of first visit registries. We could not exclude multi-partner MSM specifically recruited for the Swiss STAR trial (2016–2017), which could have resulted in selection bias. Finally, our study results are suited to our setting as the predicted trajectories are dependent on the questionnaires used in the counseling session as well as the on the social and demographic characteristics of our study population. These often differ between different regions and populations.

### Significance

At the population level, our study provides results and a novel methodological approach to frame sexual health messages. These messages might be more likely to resonate with MSM’s individual needs than those addressing broader segments of the population, and complement and improve current efforts to provide precise messages to subpopulations of MSM. At the level of individualized counseling by caregivers, our results and methodology could help contextualize individuals’ sexual behavior to the light of ongoing trends in the MSM population, and constitutes a quantitative tool for the identification of probable forthcoming behavioral changes in newly registered MSM. Unlike individualized or high-granularity approaches, the method outlined in this manuscript provides an alternative to address relevant behavioral groups that account from relatively large fractions of the population, which might favor design and dissemination of public, yet sharp messages on sexual health.

Routine update of our results characterizing the evolution in time of trends in combinations of sexual behaviors could be used to detect impacts of public health interventions at the population level, including campaigns addressing sexual behavior or substance use as well as changes in reimbursement policies for STI-testing or PrEP. Moreover, the identification of clusters with rapidly rising frequencies of group sex (which are often accompanied by Chemsex and alcohol use for sex; subgroup A and C in the case or our results) could signal the need for timely dissemination of information on sex parties for the relevant subgroups, even if no significant increases are found at the overall population level. Early estimation of the most likely cluster as proposed in this manuscript could serve as trigger for the release of customized related information. The validation of such potential intervention warrants prospective studies in diverse settings.

Moreover, these type of approaches have the potential to help dismantle biases that persist in our society, which sometimes overlook the significant heterogeneity in sexual behaviors among men who have sex with men (MSM) and other sexual minorities. Because our results illustrate the extent and dynamics of behavioral heterogeneity, they can be suitable resources for the education of, for example, medical students and counselors, thereby positively impacting caregiver-client interactions.

In summary, this machine-learning-based study evidenced heterogeneous dynamical patterns in sexual behavior among MSM masked behind overall trends. Data from first visits predicted subgroups and thereby patterns of change in sexual behavior over the study period. Our study contributes evidence to demonstrate the opportunity to further support the transition from broader representations of sexual behavior toward precise sexual behavioral partitions of the MSM population.

## Supplementary Information

Below is the link to the electronic supplementary material.

Supplementary file1 (DOCX 917 KB)

## Data Availability

According to Swiss Law, we are not entitled to share these data.

## Code Availability

Codes are available upon request by email to the authors.
