# Supplementary material for: Machine Learning Identifies Sexual Behavior Subgroups Among Men Who Have Sex with Men in Switzerland
Source: Arch Sex Behav. 2025 Jul 7;54(6):2043–55. doi: 10.1007/s10508-025-03187-2 (PMC12283876; doi:10.1007/s10508-025-03187-2)
Supplement: Supplementary file 1 — Supplementary file1 (DOCX 917 KB) [file 10508_2025_3187_MOESM1_ESM.docx]

Supplementary material

**Machine Learning Identifies Sexual Behaviour Subgroups Among Men who Have Sex with Men in Switzerland**

*Luisa Salazar-Vizcaya, el at*

**Table S1.** Summary of variables available for analyses

| **Variable** | **Possible outcomes** | **Valid records** | |  |
| --- | --- | --- | --- | --- |
|  |  | **First visit** | **Longitudinal** | **Coding for clustering** |
| Year or birth | Range: 1940–2002 | 2349 | - | - |
| Region in Switzerland of the VCT centre | Zurich region; Lake Geneva region; Other Swiss regions | 2349 | - | - |
| Nationality | Swiss (or Liechtenstein); Neighbouring country (Austria, Germany, France, Italy), African; Asian; Eastern/South-eastern European; Latin American, Spanish, Portuguese; Other Western European, US, Canada; Other; Unknown | 2349 | - | - |
| Time living in Switzerland | <1 year ; >5 years; 1-2 years; 3-5 years; Swiss | 2339 | - | - |
| Symptom of any STI | Yes; No | 2334 | - | - |
| Sexual life happiness | Rather happy; Rather unhappy; Very happy; Very unhappy | 1644 | - | - |
| HIV status of sexual partner | HIV-negative; HIV-positive; | 983 | - | - |
| Female sexual partners* | >5; >50; 1; 11-20; 2; 2-5; 21-50; 3-5; 6-10; I don't want to say; Zero | 429 | **-** | **-** |
| Number of anal sex partners* | >5; Up to 5; Zero | 2349 | 11269 | >5; Up to 5; Zero |
| Partnership status | Single; Currently in a long-term relationship; It's not yet clear | 2325 | 10081 | Single or in a stable relationship |
| Sexual partners met online | None; About half; Less than half; More than half; Nearly all of them; Yes | 2243 | 11114 | More than half, up to half; None |
| Group sex* | Yes; No | 2216 | 11048 | Yes; No |
| Condomless anal intercourse with non-steady partners (nsCAI)** | Yes; No | 2206 | 10702 | Yes; No |
| Alcohol use for sex | No large amounts alcohol for sex; Yes often or always when having sex; Yes, rarely | 1497 | 9278 | Yes = {Yes often or always when having sex}; No = {No large amounts alcohol for sex, Yes, but rarely} |
| Chemsex and other sexualised drug use, SDU)* | 4-chems (GHB, Ketamin, Mephedron, or Crystal Meth); SDU but not the 4-chems; No SDU; | 1361 | 8848 | Yes = {4-chems (GHB, Ketamin, Mephedron, or Crystal Meth); SDU but not the 4-chems}; No = {No SDU} |

*in the previous 12 months;**in the previous 3 months.

**Text S1 - Method**

*Early identification of subgroup members*

We ran the classifier independently for each of the aforementioned five levels (six counting the trivial top level) of the classification and used the area under the receiving operating curve (auc ROC) as measure of accuracy.

Because it is always possible to run classifier at all levels of the hierarchy, we hereby report the accuracy of the method to identify members of a cluster as the highest across levels *e.g.* consider a cluster that appears in the second level and remains in the third level. The classifier runs for both levels and if the accuracy for that cluster is 90% at level 2 and 88% at level 3, we report 90% accuracy.

**Figure S1. Random forest classifier ROC curves for early identification of members of subgroups of interest per level in the hierarchy.**
Colour labels correspond to the original numbering of clusters. Translation to groups of interest as shown in Figure 3 is as follows: 2355: Subgroup A, 2356: Subgroup B, 2353: C, 2354:D, 2357:D1, 2359:D2. When a subgroup of interest belonged in more than one level, we chose the best prediction (i.e. largest area under the ROC).


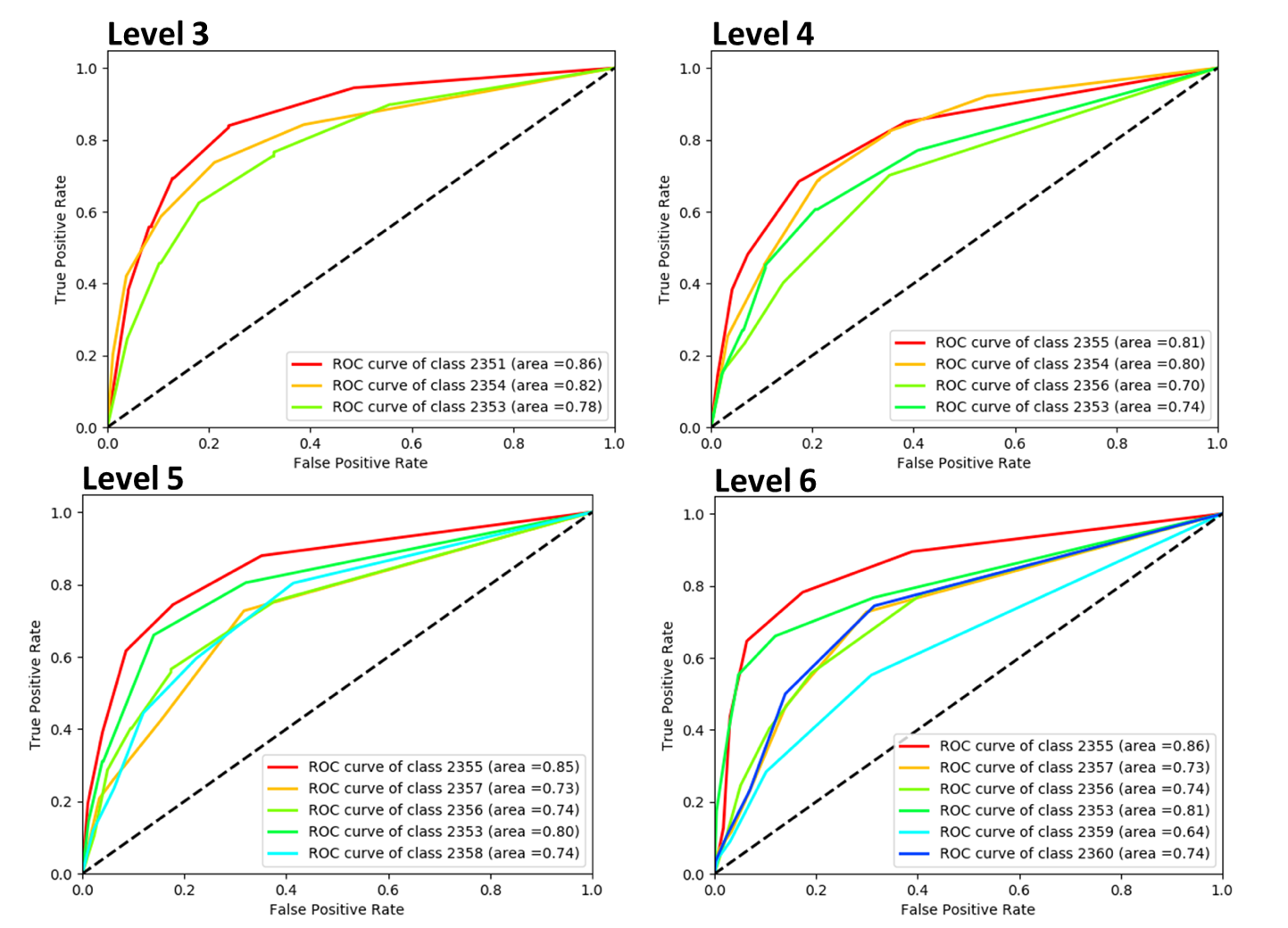


**Figure S2. Features importance in the random forest classifier for early identification of members of subgroups of interest per level in the hierarchy.**Level 3 predicts membership for subgroups of interest C and D; Level 4 for subgroups of interest A, B, C and D; Level 5 for subgroups of interest: A, B, C and D1; Level 6 for subgroups of interest: A, B, C, D1, D2. See Figure 1.

**
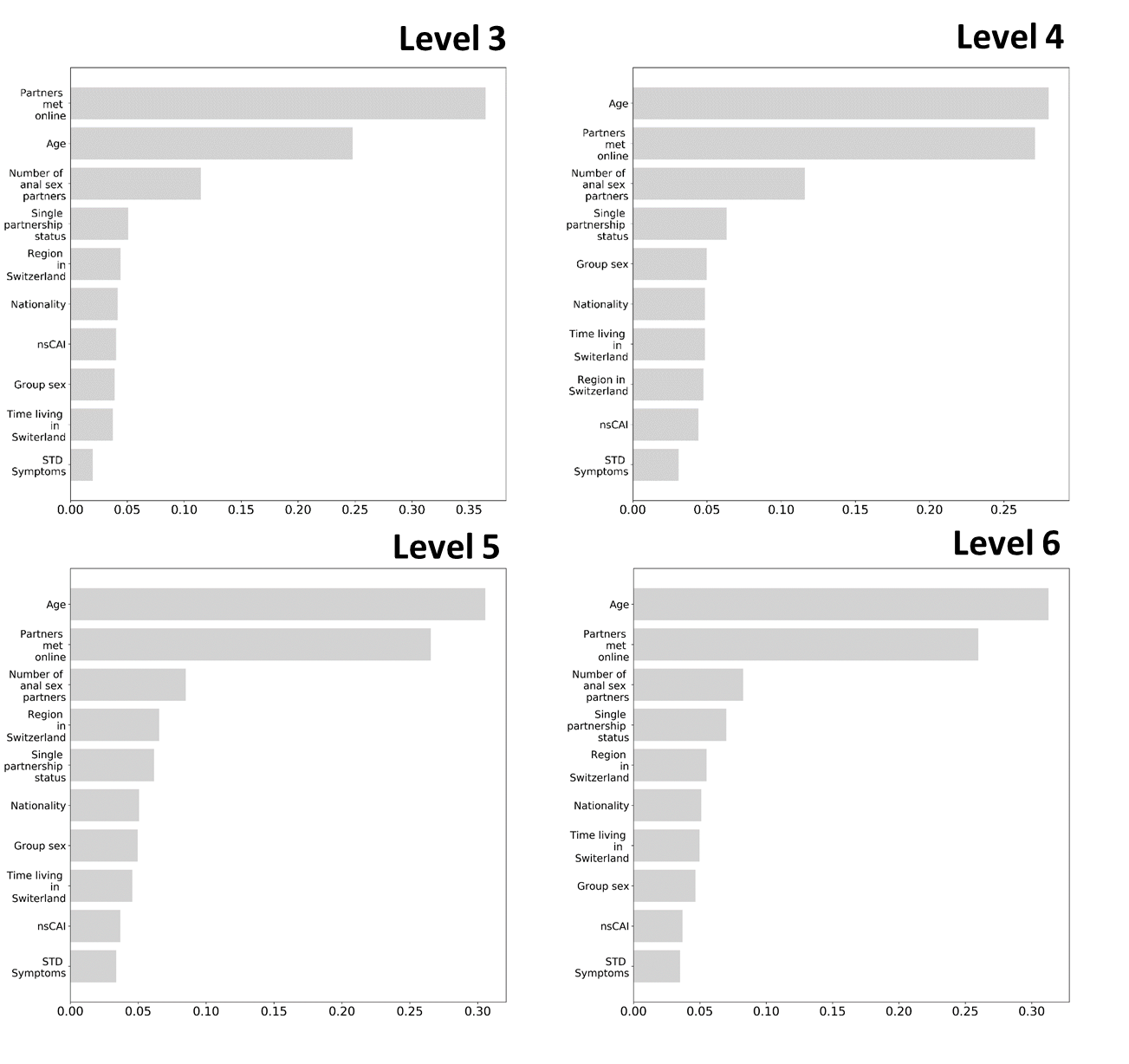
**
